# Systematic Genetic Nomenclature for Type VII Secretion Systems

**DOI:** 10.1371/journal.ppat.1000507

**Published:** 2009-10-30

**Authors:** Wilbert Bitter, Edith N. G. Houben, Daria Bottai, Priscille Brodin, Eric J. Brown, Jeffery S. Cox, Keith Derbyshire, Sarah M. Fortune, Lian-Yong Gao, Jun Liu, Nicolaas C. Gey van Pittius, Alexander S. Pym, Eric J. Rubin, David R. Sherman, Stewart T. Cole, Roland Brosch

**Affiliations:** 1 VU University Medical Centre, Amsterdam, The Netherlands; 2 Dipartimento di Patologia Sperimentale, Biotecnologie Mediche, Infettivologia ed Epidemiologia, University of Pisa, Pisa, Italy; 3 Inserm Avenir Group, Institut Pasteur Korea, Seoul, Korea; 4 Department of Microbial Pathogenesis, Genentech Inc., San Francisco, California, United States of America; 5 Department of Microbiology and Immunology, University of California, San Francisco, California, United States of America; 6 Wadsworth Center, New York State Department of Health, Albany, New York, United States of America; 7 Department of Immunology and Infectious Diseases, Harvard School of Public Health, Boston, Massachusetts, United States of America; 8 Department of Cell Biology and Molecular Genetics, University of Maryland, College Park, Maryland, United States of America; 9 Department of Molecular Genetics, University of Toronto, Toronto, Ontario, Canada; 10 Department of Biomedical Sciences, Stellenbosch University, Stellenbosch, South Africa; 11 Unit for Clinical and Biomedical TB Research, South African MRC, Durban, South Africa; 12 Seattle Biomedical Research Institute, Seattle, Washington, United States of America; 13 Global Health Institute, EPFL, Lausanne, Switzerland; 14 Institut Pasteur, Integrated Mycobacterial Pathogenomics, Paris, France; The Fox Chase Cancer Center, United States of America

Mycobacteria, such as the etiological agent of human tuberculosis, *Mycobacterium tuberculosis*, are protected by an impermeable cell envelope composed of an inner cytoplasmic membrane, a peptidoglycan layer, an arabinogalactan layer, and an outer membrane. This second membrane consists of covalently linked, tightly packed long-chain mycolic acids [1],[2] and non-covalently bound shorter lipids involved in pathogenicity [3]–[5]. To ensure protein transport across this complex cell envelope, mycobacteria use various secretion pathways, such as the SecA1-mediated general secretory pathway [6],[7], an alternative SecA2-operated pathway [8], a twin-arginine translocation system [9],[10], and a specialized secretion pathway variously named ESAT-6-, SNM-, ESX-, or type VII secretion [11]–[16]. The latter pathway, hereafter referred to as type VII secretion (T7S), has recently become a large and competitive research topic that is closely linked to studies of host–pathogen interactions of *M. tuberculosis* [17] and other pathogenic mycobacteria [16]. Molecular details are just beginning to be revealed [18]–[22] showing that T7S systems are complex machineries with multiple components and multiple substrates. Despite their biological importance, there has been a lack of a clear naming policy for the components and substrates of these systems. As there are multiple paralogous T7S systems within the Mycobacteria and orthologous systems in related bacteria, we are concerned that, without a unified nomenclature system, a multitude of redundant and obscure gene names will be used that will inevitably lead to confusion and hinder future progress. In this opinion piece we will therefore propose and introduce a systematic nomenclature with guidelines for name selection of new components that will greatly facilitate communication and understanding in this rapidly developing field of research.

The first T7S-associated protein to be identified was the 6-kD early secreted antigenic target ESAT-6 [23]. This small, highly immunogenic protein lacks a classical N-terminal signal sequence and is present in large amounts in the culture filtrate of *M. tuberculosis* [23], but is missing from the closely related attenuated live vaccine *Mycobacterium bovis* bacille Calmette-Guérin (BCG) [24] due to the deletion of region of difference 1 (RD1) [25]. ESAT-6 and its protein partner, the 10-kD culture filtrate protein CFP-10 [26], form a 1∶1 protein complex [27] that involves hydrophobic interaction [18],[28]. Secretion of ESAT-6 and CFP-10 is required for the pathogenicity of *M. tuberculosi*s [29]–[31]. The absence of ESAT-6 secretion is responsible in part for the attenuation of the BCG and *Mycobacterium microti* vaccines [13],[32],[33], as well as for the decrease in virulence of the attenuated *M. tuberculosis* H37Ra strain [34].

In *M. tuberculosis*, ESAT-6 and CFP-10 belong to the WXG100 family of 23 small secreted proteins that share a size of approximately 100 amino acids, a helical structure, and a characteristic hairpin bend formed by the conserved Trp-Xaa-Gly (W-X-G) motif [35]. The genes encoding these proteins, many of which represent immunodominant T cell antigens [36], are called *esx* genes in *M. tuberculosis* (*esxA*-*W*, Table 1) and are arranged in tandem pairs at 11 genomic loci [37]. In five of these genomic loci (ESX-1–ESX-5), the *esx* genes are flanked by genes coding for components of secretion machineries involved in the export of the corresponding ESX proteins (Figure 1). These proteins constitute the major building blocks of the T7S systems [11],[12],[15],[16],[19]. Four of these regions are also characterized by the presence of genes encoding PE and/or PPE proteins (Figure 1, Table 2), named after their characteristic N-terminal motifs proline-glutamic acid (PE) and proline-proline-glutamic acid (PPE) [38]. Apart from genes localized in these core ESX regions, additional genes situated elsewhere on the chromosome may be required for the function of T7S systems. For example, the *rv3616c-rv3614c* genes are required for secretion of ESAT-6 and CFP-10 by ESX-1 [39]–[41].

**Figure 1:**
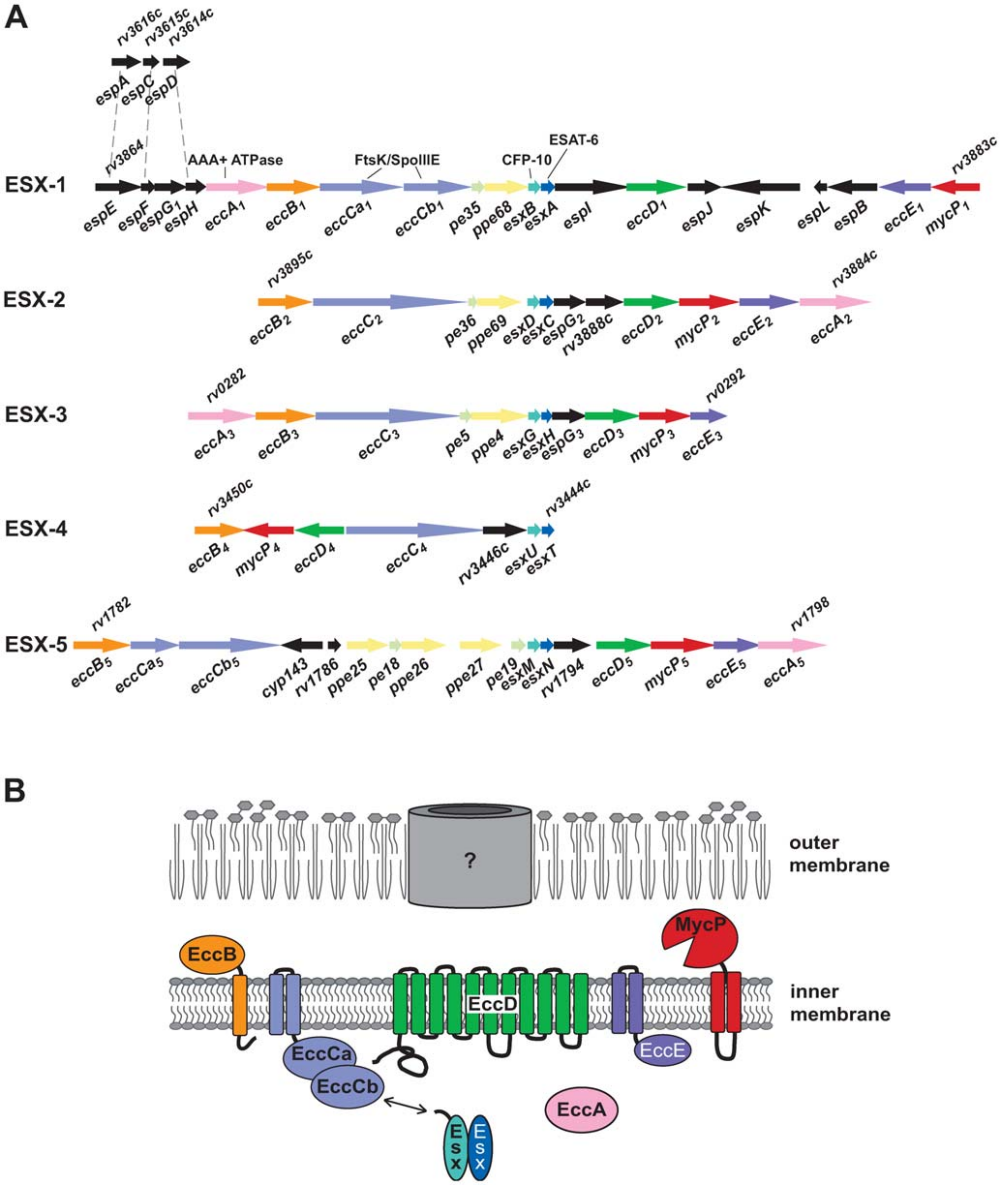
Genetic organization of the 5 ESX loci and the *espA* operon in *M. tuberculosis* H37Rv with the proposed nomenclature and predicted cellular localization of the conserved ESX gene products and their interactions. (A) Genetic organization. (B) Model. The abbreviation *ecc* stands for esx conserved component, whereas *esp* stands for ESX-1 secretion-associated proteins. The topology of the different proteins in the cytoplasmic membrane shown in (B) refers to the ESX-1 cluster and is based on predictions made using the MEMSAT3 algorithm [60]. Note that the channel drawn in the outer membrane of our model refers to a hypothetical pore, whose existence has not been experimentally demonstrated.

**Table 1:** Overview of *esx* Genes (WXG100 Family) of *M. tuberculosis* H37Rv, Also Showing Previously Used Gene Names in Brackets.

Gene Family	ESX-1	ESX-2	ESX-3	ESX-4	ESX-5	No Similarity To Cluster
ESAT-6	***esxA*** * (esat-6, rv3875)*	***esxC*** * (rv3890c)*	***esxH*** * (cfp7, tb10.4, rv0288)*	***esxT*** * (rv3444c)*	***esxN*** * (mtb9.9A, Rv1793)*	
CFP-10	***esxB*** * (lhp, cfp-10, rv3874)*	***esxD*** * (rv3891c)*	***esxG*** * (tb9.8, rv0287)*	***esxU*** * (rv3445c)*	***esxM*** * (tb11.0, rv1792)*	
ESAT-6 homologues elsewhere in the genome			***esxR*** * (tb10.3, rv3019c)*, ***esxQ*** * (tb.9, rv3017c)*		***esxI*** * (mtb9.9D, rv1037c)*, ***esxL*** * (mtb9.9C, rv1198)*, ***esxO*** * (mtb9.9E, rv2346c)*, ***esxV*** * (mtb9.9D, rv3619c)*	***esxE*** * (rv3904c)*
CFP-10 homologues elsewhere in the genome			***esxS*** * (rv3020c)*		***esxJ*** * (tb11.0, Rv1038c)*, ***esxK*** * ( tb11.0, Rv1197)*, ***esxP*** * (rv2347c)*, ***esxW*** * (rv3620c)*	***esxF*** * (rv3905c)*

**Table 2:** New and Old Nomenclature of the Different Esx Conserved Components (*ecc* Genes) and Genes Encoding ESX-1 Secretion-Associated Proteins (*esp* Genes) of the T7S Systems of *M. tuberculosis* H37Rv.

New Gene Name^a^	Putative Function of Gene Products	Previously Proposed Gene Names				
		ESX-1	ESX-2	ESX-3	ESX-4	ESX-5
***eccA***	AAA+ ATPase	*rv3868*	*rv3884c*	*rv0282*	*-*	*rv1798*
***eccB***	Transmembrane protein (1 TM)	*rv3869*	*rv3895c*	*rv0283*	*rv3450c*	*rv1782*
***eccC***	FtsK/SpoIIIE-like transmembrane protein (1–3 TMs)	*-*	*rv3894c*	*rv0284*	*rv3447c*	*-*
***eccCa***	FtsK/SpoIIIE-like transmembrane protein (1–3 TMs)	*rv3870 snm1*	*-*	*-*	*-*	*rv1783*
***eccCb***	FtsK/SpoIIIE-like transmembrane protein (1–3 TMs)	*rv3871 snm2*	*-*	*-*	*-*	*rv1784*
***eccD***	Transmembrane protein (10–11 TMs)	*rv3877 snm4*	*rv3887c*	*Rv0290*	*rv3448c*	*rv1795*
***eccE***	Transmembrane protein (2 TMs)	*rv3882c*	*rv3885c*	*rv0292*	*-*	*rv1797*
***mycP***	Subtilisin-like serine protease (mycosin) (1 TM)	*rv3883c*	*rv3886c*	*rv0291*	*rv3449c*	*rv1796*
***espA***	Secreted protein	*rv3616c*	*-*	*-*	*-*	*-*
***espB***	Secreted protein	*rv3881c*	*-*	*-*	*-*	*-*
***espC***	Secreted protein	*rv3615c*	*-*	*-*	*-*	*-*
***espD***	Unknown	*rv3614c*	*-*	*-*	*-*	*-*
***espE***	Secreted protein	*rv3864*	*-*	*-*	*-*	*-*
***espF***	Secreted protein	*rv3865*	*-*	*-*	*-*	*-*
***espG***	Soluble protein	*rv3866*	*rv3889c*	*rv0289*	*-*	*-*
***espH***	Unknown	*rv3867*	*-*	*-*	*-*	*-*
***espI***	Pro and Ala rich protein	*rv3876 snm3*	*-*	*-*	*-*	*-*
***espJ***	Unknown	*rv3878*	*-*	*-*	*-*	*-*
***espK***	Pro and Ala rich protein	*rv3879c*	*-*	*-*	*-*	*-*
***espL***	Unknown	*rv3880c*	*-*	*-*	*-*	*-*
***espR***	Regulation	*rv3849*	*-*	*-*	*-*	*-*

The number of transmembrane domains varies depending on the prediction programme used (for details see Table S2).

a The numeral suffix indicating the ESX cluster to which this gene belongs is not shown in this table.

Apart from members of the *M. tuberculosis* complex, the ESX-1 cluster is also present in a range of mycobacteria, including *Mycobacterium kansasii* [23] and *Mycobacterium leprae* [42]. However, experimental work has mainly focused on the ESX-1 system of *Mycobacterium marinum* [21], [22], [43]–[47], a fish pathogen that shows high homology in its ESX loci with *M. tuberculosis* [48], and the fast grower *Mycobacterium smegmatis* [49]–[51]. *M. marinum* has also been used to define a role for the paralogous system ESX-5, which is required for the secretion of PE and PPE proteins [16],[52],[53]. For the remaining ESX-2, ESX-3, and ESX-4 systems, only very limited predictions of their putative functions can be made. ESX-3 transcriptome data suggest that this system is involved in iron/zinc homeostasis [54],[55], which would be consistent with the essential role of ESX-3 in *M. tuberculosis* [56]. The putative functions of ESX-2 and ESX-4 remain unknown. ESX-4, which harbors a smaller number of genes than other ESX loci (Table 2), appears to represent the most ancestral T7S system in mycobacteria [12]. This hypothesis is based on the observation that ESX-4-like loci are the only ESX clusters that are found in other high GC Gram-positive bacteria, suggesting that the last common ancestor of mycobacteria already harbored an ESX-4 T7S system. Other ESX clusters may have evolved later by gene duplication and gene diversification events. However, the finding that *Nocardia farcinica* (http://nocardia.nih.go.jp/) contains two T7S systems, one orthologous to ESX-4 and one locus that shows some similarity to all the conserved components of larger T7S systems, suggests that evolution of T7S systems is more complex than previously anticipated. This second T7S locus in *N. farcinica* even contains two PPE-like genes that were originally thought to be specific for the mycobacteria [38].

T7S-like systems are also found outside the high GC Gram-positive bacteria, since a number of Firmicutes have WXG100 members [35]. However, the loci containing these WXG100 genes are only weakly similar to the mycobacterial T7S systems: in fact, only the gene encoding the FtsK/SpoIIIE-like protein is present. Therefore, these systems should be called WXG100 systems to differentiate them from true T7S systems. Both *Staphylococcus aureus* and *Bacillus anthracis* have an active WXG100 system, and the WXG100 system encoded by *S. aureus* is important for virulence [57],[58].

Research in the T7S/ESX field is relatively new, but is now rapidly expanding and we therefore would like to propose a systematic nomenclature for all components involved. Until now a small number of genes within the different ESX loci of mycobacteria have been named, but for most genes the original genome annotation numbers are used. These gene numbers vary between different species and even between different strains of the same species, and therefore make comparative studies confusing. Our nomenclature is appropriate for all T7S systems in high GC-Gram-positive species. Extending this nomenclature to the T7S-like systems of Firmicutes is not recommended, since there are only a very few conserved components.

As a starting point for the new nomenclature, we focus on the most studied system, the ESX-1 system of *M. tuberculosis*, which is the paradigm T7S system. The new nomenclature is given for ESX-1 in *M. tuberculosis* (Figure 1 and Table 2) and for all ESX systems in various Mycobacteria (Table S1). The proposed rules for the nomenclature are as follows:

- Only genes that have homologues in at least four of the mycobacterial ESX systems will get a general name, whereas the locus-specific genes have a more restricted name reflecting their specificity. The reason for this distinction is that the conserved genes are most likely to represent the core components of the secretion system. Moreover, all of the conserved ESX-1 components have been shown to be essential for ESAT-6/CFP-10 secretion in at least one of the mycobacterial species studied (See below). In contrast, many of the locus-specific genes encode secreted proteins, as has been shown for the ESX-1 system (see below). Furthermore, in *M. leprae*, an organism with an extreme reductive evolution of its genome, almost all of the non-conserved ESX-1 components are pseudogenes, whereas all of the conserved components seem to be intact [42].
- The three letter acronym for the conserved components will be *ecc*, for esx conserved component (Figure 1, Table 2). This abbreviation has not been used for other genes in bacteria.
- The ESAT-6 and CFP-10 encoding genes, *esxA* and *esxB*, respectively, and the other *esx* genes (Table 1) will not be renamed. These gene names are informative, well-accepted, and frequently used in the literature. Furthermore, the *esx* gene products seem to be secreted proteins and do not seem to be components of the secretion system itself, although their presence is required for the secretion of other substrates. The same reasoning is used for the *pe* and *ppe* genes. Four of the five systems harbor *pe* and *ppe* genes, but for the moment their functions within the T7S systems remain uncertain. Furthermore, various mycobacterial species contain a large number of genes belonging to the *pe* and *ppe* families, and it would be confusing to rename some of them. Finally, the subtilisin-like proteases already have an established and descriptive name in literature, i.e., the mycosins [59]. Therefore, we will not change this name.
- The alphabetic suffix of conserved genes will be based on the gene order in the paradigm ESX-1 system (see Figure 1). This decision is mainly based on the fact that the ESX-1 system is the most studied. The gene order of the different T7S systems is highly variable and it is therefore difficult to propose a logical ordering that would be satisfactory for all systems. The genes of ESX-2/-3/-4 and -5 will therefore be named according to their paralogue in ESX-1 (Table 2 and Table S1), allowing for a direct and relevant comparison. The gene names of each mycobacterial T7S will include a numeral suffix indicating the ESX cluster to which this gene belongs. In order to avoid confusion with numbering of alleles, the ESX cluster number is indicated in subscript. As shown in Figure 1, the first conserved gene of the ESX-1 cluster will be *eccA_1_*.
- In some of the T7S clusters, the gene encoding the FtsK/SpoIIIE-like protein is split in two genes. Since these gene products clearly form a functional unit, as has also been shown for the two FtsK/SpoIIIE-like proteins of the ESX-1 system [14], the split genes will get a lower case alphabetic suffix, i.e., *eccCa_1_* and *eccCb_1_* for the ESX-1 system of *M. tuberculosis* (Figure 1 and Table 2).
- When working with several different organisms, it can also be useful to indicate the origin of the respective genes. For this we recommend using a two-letter subscript at the end of the gene name. For example, the orthologues of the *M. tuberculosis* genes *eccCa_1mt_* and *eccCb_1mt_* would be *eccCa_1ms_* and *eccCb_1ms_* in *M. smegmatis*.
- The gene names can be converted into their proteins by capitalization, e.g., EccCa_1_. Alternatively, once the true function of a protein is known, the name could be changed to indicate this function, as has been done for the secretins of type II and type III secretion systems. If in the future new genes are identified that are essential for the functioning of several T7S systems, these genes could be named similarly using the next alphabetical suffix (*eccG*, *eccH*, etc.).
- As discussed above, in addition to the conserved genes, there are also region-specific genes. The role of these genes in ESAT-6/CFP-10 secretion is not entirely clear: some of the encoded proteins seem to be involved in the secretion of T7S substrates in *M. marinum*, whereas their orthologues show less or no effect on secretion in *M. tuberculosis*. Recently, it has been shown that a subset of these proteins are in fact also substrates of the ESX-1 system. Thus far, four ESX-1 substrates have been identified in addition to ESAT-6 and CFP-10. These substrates are called EspA [39], EspB [46], EspR [41], and the *M. marinum* homologue of Rv3864 [22]. The acronym Esp stands for ESX-1 secretion-associated protein. Both *rv3864* and *espB* are located within the ESX-1 cluster, whereas EspA and the secreted regulatory protein EspR are encoded by genes outside the ESX-1 locus. However, the *espA* gene is part of an operon (*rv3616-3614*) that has paralogues in the 5′ region of the ESX-1 locus. Therefore, we propose naming all the region-specific genes of the ESX-1 system and the rest of the *espA* operon *esp* genes with alphabetical suffixes (see Table 2 and Figure 1). We will follow the *espA* operon and ESX-1 gene order, with the exception of *espB* and *espR*, which are already named. This means that the first gene in the *esx-1* operon, whose gene product was recently shown to be secreted protein in *M. marinum*, will be named *espE*. One of the new *esp* genes, *espG*, is present with low but significant homology in two other ESX systems (ESX-2 and ESX-3) and should therefore also have a numeral suffix (Figure 1, Table 2).
- The nomenclature of *esp* genes in *M. marinum* is more complicated, in particular for *espA*. The genome of *M. marinum* contains a large gene cluster upstream of the ESX-1 locus, among which are 15 *espA*-like genes [48]. In addition, there are three more paralogues at other locations in the genome. These genes should all be named *espA* with a superscript numeral suffix to indicate the exact gene and a subscript “*mm*” to indicate the species.
- Region-specific genes or genes encoding secreted proteins of the other ESX loci and T7S systems should not be called *esp*, as this name should be reserved for ESX-1 related genes. If there are important region-specific genes for ESX-2/-3/-4 or -5, a new name has to be introduced.

In order to ensure wide visibility for this new nomenclature it will be included in the most extensively used mycobacterial genome databases. As a first step, selected genome browsers available at the Institut Pasteur (http://genolist.pasteur.fr/) and/or the Ecole Polytechnique Federale de Lausanne (http://tuberculist.epfl.ch/) will adopt these new rules; other databases could follow this example.

In conclusion, we would like to emphasize that the introduction of a uniform gene nomenclature for other secretion systems in Gram-negative bacteria (type II, type III) has facilitated comparative analysis of these systems. We anticipate that the acceptance/implementation of this proposal will provide similar advantages for the T7S systems.

## Supporting Information

Table S1 New and old nomenclature of the different conserved components of the T7S systems in selected mycobacteria (*M. tuberculosis* H37Rv, *M. marinum* M, *M. smegmatis* mc^2^155, *M. leprae* TN, *M. avium paratuberculosis* K10). The numeral suffices to indicate the ESX clusters to which the genes belong are omitted. Note that the ESX-2 genes of *M. avium paratuberculosis* are located in two separate genomic loci. TM, transmembrane domain.
(0.09 MB DOC)

Table S2 Comparison of the transmembrane topologies and signal sequence predictions of the *M. tuberculosis* H37Rv Ecc membrane proteins. Amongst the different topology prediction programs that were used (TMHMM Server v. 2.0, MEMSAT3, Philius, SCAMPI, HMMTOP and Phobius) MEMSAT3 gave the correct prediction for the highest number of Ecc membrane proteins. Therefore, only the topology prediction results of TMHMM (used on the TubercuList server) and MEMSAT3 are shown. The clearly incorrect predictions are depicted in gray. TM, transmembrane domain; in, cytoplasmic location; out, periplasmic location; C, C-terminus; N, N-terminus; ss, signal sequence.
(0.07 MB DOC)
