# Supplementary material for: Systematic Genetic Nomenclature for Type VII Secretion Systems
Source: PLoS Pathog. 2009 Oct 30;5(10):e1000507. doi: 10.1371/journal.ppat.1000507 (PMC2763215; doi:10.1371/journal.ppat.1000507)
Supplement: Table S1 — New and old nomenclature of the different conserved components of the T7S systems in selected mycobacteria (M. tuberculosis H37Rv, M. marinum M, M. smegmatis mc2155, M. leprae TN, M. avium paratuberculosis K10). The numeral suffices to indicate the ESX clusters to which the genes belong are omitted. Note that the ESX-2 genes of M. avium paratuberculosis are located in two separate genomic loci. TM, transmembrane domain. (0.09 MB DOC) [file ppat.1000507.s001.doc]

**Suppl. Table 1.** New and old nomenclature of the different conserved components of the T7S systems in selected mycobacteria (*M. tuberculosis* H37Rv, *M. marinum* M, *M. smegmatis* mc^2^155, *M. leprae* TN, *M. avium paratuberculosis* K10). The numeral suffices to indicate the ESX clusters to which the genes belong are omitted. Note that the ESX-2 genes of *M. avium paratuberculosis* are located in two separate genomic loci. TM, transmembrane domain.

| **New gene name** | **ESX-1** | **ESX-2** | **ESX-3** | **ESX-4** | **ESX-5** |
| --- | --- | --- | --- | --- | --- |
| ***eccA* AAA+ ATPase** | | | | | |
| *eccA_mt_* | *rv3868* | *rv3884c* | *rv0282* | *-* | *rv1798* |
| *eccA_mm_* | *mmar_5443* | *-* | *mmar_0541* | *-* | *mmar_2680* |
| *eccA_ms_* | *msmeg_0059* | *-* | *msmeg_0615* | *-* | *-* |
| *eccA_ml_* | *ml0055c* | *-* | *ml2537c* | *-* | *ml1536c* |
| *eccA_map_* | *-* | *map0167* | *map3778* | *-* | *map1513* |
|  |  |  |  |  |  |
| ***eccB* Transmembrane protein (1 TM)** | | | | | |
| *eccB_mt_* | *rv3869* | *rv3895c* | *rv0283* | *rv3450c* | *rv1782* |
| *eccB_mm_* | *mmar_5444* | *-* | *mmar_0542* | *mmar_1099* | *mmar_2664* |
| *eccB_ms_* | *msmeg_0060* | *-* | *msmeg_0616* | *msmeg_1534* | *-* |
| *eccB_ml_* | *ml0054c* | *-* | *ml2536c* | *-* | *ml1544c* |
| *eccB_map_* | *-* | *map4323c**  *map4322c** | *map3779* | *map4238* | *map1501* |
|  |  |  |  |  |  |
| ***eccC* FtsK/SpoIIIE-like transmembrane protein (2 TMs)** | | | | | |
| *eccC_ms_*  *eccCa_ms_*  *eccCb_ms_* | *-*  *rv3870*  *rv3871* | *rv3894c*  *-* | *rv0284*  *-* | *rv3447c*  *-* | *-*  *rv1783*  *rv1784* |
| *eccC_mm_*  *eccCa_mm_*  *eccCb_mm_* | *-*  *mmar_5445*  *mmar_5446* | *-* | *mmar_0543*  *-* | *mmar_1102*  *-* | *mmar_2665*  *-* |
| *eccC_ms_*  *eccCa_ms_*  *eccCb_ms s_* | *-*  *msmeg_0061*  *msmeg_0062* | *-* | *msmeg_0617*  *-* | *msmeg_1536*  *-* | *-* |
| *eccC_ml_*  *eccCa_ml_*  *eccCb_ml_* | *-*  *ml0053c*  *ml0052c* | *-* | *ml2535c*  *-* | *-* | *ml1543c*  *-* |
| *eccC_map_* | *-* | *map4321c* | *map3780* | *map4241* | *map1502* |
|  |  |  |  |  |  |
| ***eccD* Transmembrane protein (11 TMs)** | | | | | |
| *eccD_mt_* | *rv3877* | *rv3887c* | *rv0290* | *rv3448* | *rv1795* |
| *eccD_mm_* | *mmar_5452* | *-* | *mmar_0549* | *mmar_1101* | *mmar_2677* |
| *eccD_ms_* | *msmeg_0068* | *-* | *msmeg_0623* | *msmeg_1535* | *-* |
| *eccD_ml_* | *ml0047c* | *-* | *ml2529c* | *-* | *ml1539c* |
| *eccD_map_* | *-* | *map0164* | *map3786* | *map4240c* | *map1510* |
|  |  |  |  |  |  |
| ***eccE* Transmembrane protein (2 TMs)** | | | | | |
| *eccE_mt_* | *rv3882c* | *rv3885c* | *rv0292* | *-* | *rv1797* |
| *eccE_mm_* | *mmar_5458* | *-* | *mmar_0551* | *-* | *mmar_2679* |
| *eccE_ms_* | *msmeg_0082* | *-* | *msmeg_0626* | *-* |  |
| *eccE_ml_* | *ml0042* | *-* | *ml2527c* | *-* | *ml1537c* |
| *eccE_map_* | *-* | *-* | *map3788* | *-* | *map1512* |
| ***eccF* Subtilisin-like serine protease (Mycosin) (1-2 TMs)** | | | | | |
| *mycP_mt_* | *rv3883c* | *rv3886c* | *rv0291* | *rv3449* | *rv1796* |
| *mycP_mm_* | *mmar_5459* | *-* | *mmar_0550* | *mmar_1100* | *mmar_2678* |
| *mycP_ms_* | *msmeg_0083* | *-* | *msmeg_0624* | *msmeg_1533* | *-* |
| *mycP_ml_* | *ml0041* | *-* | *ml2528c* | *-* | *ml1538c* |
| *mycP_map_* | *-* | *map0165* | *map3787* | *map4239c* | *map1511* |
|  |  |  |  |  |  |
| ***espG* Soluble protein** | | | | | |
| *espG_mt_* | *rv3866* | *rv3889c* | *rv0289* | *-* | *-* |
| *espG_mm_* | *mmar_5441* | *-* | *mmar_0548* | *-* | *-* |
| *espG_ms_* | *msmeg_0057c* | *-* | *msmeg_0622* | *-* | *-* |
| *espG_ml_* | *pseudogene ml0057* | *-* | *ml2530c* | *-* | *-* |
| *espG_map_* | *-* | *map0162* | *map3785* | *-* | *-* |
|  |  |  |  |  |  |

* *eccB_2_*in *M. paratuberculosis* is split up in two genes.

Genome data information was retrieved from following websites:

*M. tuberculosis* H37Rv: <http://genolist.pasteur.fr/TubercuList/>

*M. marinum* M: <http://genolist.pasteur.fr/MarinoList/>

*M. smegmatis* mc2 155: <http://www.ncbi.nlm.nih.gov/sites/entrez?db=genomeprj&cmd=Retrieve&dopt=Overview&list_uids=92>

*M. leprae* TN*:* <http://genolist.pasteur.fr/Leproma/>

*M. avium paratuberculosis* K10: <http://www.ncbi.nlm.nih.gov/sites/entrez?db=genomeprj&cmd=Retrieve&dopt=Overview&list_uids=91>
