# Supplementary material for: Systematic Genetic Nomenclature for Type VII Secretion Systems
Source: PLoS Pathog. 2009 Oct 30;5(10):e1000507. doi: 10.1371/journal.ppat.1000507 (PMC2763215; doi:10.1371/journal.ppat.1000507)
Supplement: Table S2 — Comparison of the transmembrane topologies and signal sequence predictions of the M. tuberculosis H37Rv Ecc membrane proteins. Amongst the different topology prediction programs that were used (TMHMM Server v. 2.0, MEMSAT3, Philius, SCAMPI, HMMTOP and Phobius) MEMSAT3 gave the correct prediction for the highest number of Ecc membrane proteins. Therefore, only the topology prediction results of TMHMM (used on the TubercuList server) and MEMSAT3 are shown. The clearly incorrect predictions are depicted in gray. TM, transmembrane domain; in, cytoplasmic location; out, periplasmic location; C, C-terminus; N, N-terminus; ss, signal sequence. (0.07 MB DOC) [file ppat.1000507.s002.doc]

**Suppl. Table 2.** Comparison of the transmembrane topologies and signal sequence predictions of the *M. tuberculosis* H37Rv Ecc membrane proteins. Amongst the different topology prediction programs that were used (TMHMM Server v. 2.0, MEMSAT3, Philius, SCAMPI, HMMTOP and Phobius) MEMSAT3 gave the correct prediction for the highest number of Ecc membrane proteins. Therefore, only the topology prediction results of TMHMM (used on the TubercuList server) and MEMSAT3 are shown. The clearly incorrect predictions are depicted in gray. TM, transmembrane domain; in, cytoplasmic location; out, periplasmic location; C, C-terminus; N, N-terminus; ss, signal sequence.

|  | **TMHMM** | **MEMSAT3** | **ss by SignalP-HMM?** |
| --- | --- | --- | --- |
| **EccB1** | 1 TM, C out | 1 TM, C out | no |
| **EccB2** | 1 TM, C out | 1 TM, C out | no |
| **EccB3** | 1 TM, C out | 1 TM, C out | no |
| **EccB4** | 1 TM, C out | 1 TM, C out | no |
| **EccB5*** | 2 TMs, C out | 1 TM, C out | no |
| **EccCa_1_** | 3 TMs, ATPase domain out | 2 TMs, ATPase domain in | no |
| **EccC_2_** | 2 TMs, ATPase domain out | 2 TMs, ATPase domain in | no |
| **EccC_3_** | 1 TM, ATPase domain out | 2 TMs, ATPase domain in | no |
| **EccC_4_** | 2 TMs, ATPase domain out | 3 TMs, ATPase domain in | no |
| **EccCa_5_** | 2 TMs, ATPase domain out | 1 TM, ATPase domain out | no |
| **EccD_1_** | 11 TMs, N in | 11 TMs, N in | no |
| **EccD_2_** | 11 TMs, N in | 11 TMs, N in | no |
| **EccD_3_** | 11 TMs, N in | 11 TMs, N in | no |
| **EccD_4_** | 11 TMs, N in | 11 TMs, N in | no |
| **EccD_5_** | 10 TMs, N in | 11 TMs, N in | no |
| **EccE_1_** | 2 TMs, C out | 2 TMs, C in | no |
| **EccE_2_** | 2 TMs, C out | 2 TMs, C in | yes |
| **EccE_3_** | 2 TMs, C in | 2 TMs, C in | no |
| **EccE_5_** | 2 TMs, C in | 2 TMs, C in | yes/no^ |
| **MycP_1_^*^** | 1 TM, protease domain out | ss, 2 TMs, protease domain out | yes |
| **MycP_2_^*^** | 1 TM, protease domain out | 3 TMs, protease domain out^#^ | yes |
| **MycP_3_^*^** | 1 TM, protease domain out | 2 TMs, protease domain out | yes |
| **MycP_4_^*^** | 2 TMs, protease domain out^#^ | 1 TM, protease domain out | yes |
| **MycP_5_^*^** | 2 TMs, protease domain out^#^ | 2 TMs, protease domain out^ | yes |

* It is uncertain whether EccB_5_ and the mycosins have 1 or 2 TMs.

# The first predicted TM is most likely a signal sequence.

^ It is uncertain whether EccE_5_ has a signal sequence.
